# Posttraumatic stress disorder (PTSD) in children after paediatric intensive care treatment compared to children who survived a major fire disaster

**DOI:** 10.1186/1753-2000-2-9

**Published:** 2008-05-20

**Authors:** Madelon B Bronner, Hendrika Knoester, Albert P Bos, Bob F Last, Martha A Grootenhuis

**Affiliations:** 1 Psychosocial Department, Emma Children's Hospital Academic Medical Center, University of Amsterdam, The Netherlands; 2 Department of Paediatric Intensive Care, Emma Children's Hospital Academic Medical Center, University of Amsterdam, The Netherlands; 3 Department of Developmental Psychology, Vrije Universiteit, Amsterdam, The Netherlands

## Abstract

**Background:**

The goals were to determine the presence of posttraumatic stress disorder (PTSD) in children after paediatric intensive care treatment, to identify risk factors for PTSD, and to compare this data with data from a major fire disaster in the Netherlands.

**Methods:**

Children completed the Dutch Children's Responses to Trauma Inventory at three and nine months after discharge from the paediatric intensive care unit (PICU). Comparison data were available from 355 children survivors who completed the same questionnaire 10 months after a major fire disaster.

**Results:**

Thirty-six children aged eight to 17 years completed questionnaires at three month follow-up, nine month follow-up, or both. More than one third (34.5%) of the children had subclinical PTSD, while 13.8% were likely to meet criteria for PTSD. Maternal PTSD was the strongest predictor for child PTSD. There were no significant differences in (subclinical) PTSD symptoms either over time or compared to symptoms of survivors from the fire disaster.

**Conclusion:**

This study shows that a considerable number of children have persistent PTSD after PICU treatment. Prevention of PTSD is important to minimize the profound adverse effects that PTSD can have on children's well-being and future development.

## Background

In children, posttraumatic stress disorder (PTSD) is characterized by [1] persistent reliving or remembering of the stressful event in vivid memories, repetitive play, and nightmares; [2] avoidance of thoughts or places associated with the stressful event; [3] symptoms of increased arousal, such as sleeping and concentration problems accompanied by physical symptoms and/or [4] new fears, aggressive behaviour and loss of previously acquired developmental skills. At a later stage, comorbidities (e.g., anxiety, substance abuse and depression disorder) may occur [1,2]. When not properly diagnosed and treated, PTSD may, even at subclinical levels, result in substantial impairment of social and academic functioning [3].

Diagnosis of PTSD in children was officially established in the publication of DSM-III in 1980 [4]. Ever since, the list of potential stressful events leading to PTSD during childhood has increased [5]. In 1994, both injury and being diagnosed with a life-threatening illness were listed as potential stressful events [6,7]. This resulted in an increasing number of studies examining the prevalence and risk factors of PTSD in paediatrics, predominantly in paediatric oncology and trauma patients [7,8]. The reported prevalence of PTSD varies between 5% and 35% depending on the population studied [9-11].

Within childhood PTSD, some patterns of findings do emerge. First, some studies suggest that characteristics of the medical event may play a role in the development of PTSD: [1] Prevalence of PTSD is increased after acute admissions to the paediatric intensive care unit (PICU) compared to general wards [12,13]; [2] prevalence of PTSD is increased after unexpected and life-threatening accidents compared to chronic diseases such as diabetes [14,15]; [3] children suffering from serious illnesses who are exposed to a high number of invasive procedures and a longer duration of hospital stay are more at risk for developing PTSD and psychiatric symptoms [16-18]. Second, characteristics of the child may play a prominent role in the development of PTSD: [1] Female gender and younger age at time of trauma are potential risk factors [19]; [2] psychological vulnerability may play a role as a history of exposure to stressful events and premorbid problems are predictive for PTSD [5,16,20,21]. Third, characteristics of the family may play a role in the development of PTSD. For example, parental stress reactions and coping style predict PTSD in the child [21-26].

Although, some patterns of childhood PTSD have been recognized so far, these can not solely account for the different prevalence rates reported in paediatric populations. Studies often differ in terms of study sample, in methods, and in timing of assessment. To determine the exact prevalence and the natural time course of PTSD, longitudinal studies are essential [27]. Few longitudinal studies of PTSD in paediatric populations have been completed. Furthermore, most research has been performed in hospital-based settings, whereas a limited number of studies have made comparisons between distinct stressful events [5]. These comparisons would presumably allow us to improve understanding of PTSD in paediatrics. Up to now, it is not clear whether children who are exposed to paediatric intensive care treatment display similar PTSD reactions to children who are exposed to other types of stressful events, such as a major fire disaster.

In order to gain more insight into PTSD in children after paediatric intensive care treatment, a follow-up study in our PICU was designed. In addition, we compared the data from this study to data from another study on a major fire disaster in the Netherlands. We expected children after paediatric intensive care treatment to be at risk for developing PTSD. The main research questions were: [1] What is the prevalence of PTSD in children at three and nine months follow-up after paediatric intensive care treatment? [2] How does the prevalence of PTSD at nine months after discharge from PICU relate to the prevalence of PTSD after having survived a major fire disaster in the Netherlands? [3] To what extent is the development of PTSD at nine months after discharge from the PICU influenced by the nature of the medical event, the specific characteristics of the child, and by parental stress reactions?

## Methods

### The project and study sample

This is a prospective follow-up study at three and nine months after an unexpected PICU admission, focusing on physical and psychological consequences in children and their parents. In this study, we included *previously healthy *children, *unexpectedly *referred to the PICU with an acute life-threatening medical event; we excluded children with known underlying illnesses or patients after elective surgery. In an attempt to include seriously ill patients only, we defined our inclusion criteria as admissions for respiratory insufficiency necessitating ventilatory support for at least 24 hours and/or patients admitted to the PICU for at least 7 days, including all trauma types. Exclusion criteria were admission due to abuse or self-intoxication and the inability to complete Dutch questionnaires. The study was conducted from December 2002 to October 2005. The present report will only show data on children older than eight years since the outcome measure was self-report and validated for children between the ages of eight and eighteen.

The term *previously healthy *was defined as having no need of medical supervision at any time before PICU admission. *Unexpected admission *was defined as an unplanned PICU admission due to a life-threatening medical event. This included children presenting at the emergency room and directly admitted to the PICU, as well as children first admitted to the general ward, whose condition then deteriorated and who subsequently were admitted to the PICU.

### Procedure and participants

After discharge from the PICU, each family received a letter at home explaining the aim and content of the research program. Families were then contacted by telephone to invite participation in the research program. For cases in which no telephone contact was made following repeated attempts, follow-up letters were sent with a tear-off reply slip inviting participation. Families who declined to participate were asked about their reasons for refusal. Participation in the research program included a visit to the follow-up clinic at three months and a completion of questionnaires at three and then nine months. The visit to the follow-up clinic at three months consisted of a structured medical examination of the child by a physician at PICU followed by a psychological screening by a psychologist. Prior to this clinic visit, parents and children were to complete the questionnaires at home and bring them to this screening. Some parents did not visit the follow-up clinic and only completed the questionnaires (for example, for geographical reasons). At nine months after discharge, parents and children were sent identical questionnaires as at three months. Written informed consent was obtained from all participating families. The Medical Ethics Committee of the Academic Medical Centre in Amsterdam approved the study protocol.

Between December 2002 and October 2005, 63 children were older than eight years and eligible for participation in the present study. In total, 36 (57.1%) children completed one or both questionnaires. Twenty-seven children completed none of the questionnaires. Six children and their families refused to participate. The most common reasons given for refusal included the following: 'everything is going well', 'we have seen too many hospitals', 'we need some rest' and 'we don't want to remember that time'. Twenty-one families said that they would like to participate but either never returned their questionnaires, or did not complete fully the questionnaires. No significant differences in patient characteristics were found between participants and non-participants except for gender (χ^2 ^= 3.87, df = 1, p = 0.05). Less boys than girls participated in the study (Table 1).

**Table 1:** Patient characteristics of participants and non-participants

	Participants (n = 36)	Non-participants (n = 27)	
	Median (Range)	Median (Range)	p

Age of child (yrs)	11.9 (8.0–17.1)	13.6 (8.0–17.3)	0.99
Length of stay in PICU (days)	3.0 (1.0–51.0)	3.0 (1.0–26.0)	0.76
Length of artificial ventilation (days)	1.0 (0.0–49.0)	1.0 (0.0–14.0)	0.84
Risk of mortality, PIM2 (%)	4.2 (0.2–26.6)	3.8 (0.4–80.7)	0.97

	n (%)	n (%)	

Gender of child			0.05*
Female	21 (58.3)	9 (33.3)	
Male	15 (41.7)	18 (66.7)	
Artificial ventilation			0.76
Yes	24 (66.7)	19 (70.4)	
No	12 (33.3)	8 (29.6)	
Reason for PICU admission			0.07
Trauma	23 (63.9)	11 (40.7)	
Non-trauma	13 (36.1)	16 (59.3)	

*p < 0.05. ** p < 0.01.

PICU = Paediatric Intensive Care Unit

### Comparison group

On New Year's Eve 2001, a café fire in a popular club in Volendam, The Netherlands, resulted in the worst mass burn incident in recent Dutch history. Almost 200 children had to be hospitalised; 14 of them died. In total, 36 hospitals in three countries participated in the care of the children. The disaster had a great effect on the local community. The Dutch public at large considered the disaster a national tragedy and a screening project was founded to detect psychological sequelae in survivors [28-30]. After ten months, all children at two schools in Volendam were administered the Dutch Children's Responses to Trauma Inventory [31]. Data of 1514 children were available. We only used data from 355 children that actually witnessed and/or survived the disaster, 180 girls and 175 boys with an average age of 15.2 (SD = 1.7, range 11–19).

### Measures

Posttraumatic stress in these children was measured with the Dutch Children's Responses to Trauma Inventory (CRTI), a 26-item self-report questionnaire for children between the ages of eight and eighteen [31]. The questionnaire covers 3 subscales (intrusion, avoidance, hyperarousal) according to the diagnostic DSM-IV symptoms of PTSD and one subscale for non-specific reactions. The items are rated on a three-point scale: 3 = yes; 2 = slightly; 1 = no. The total score of symptoms of PTSD, which can range from 26 to 78, can be used as an overall index of a child's stress reaction following a stressful event. Total scores between 38 and 46 indicate serious symptoms of PTSD and suggest a need for further professional support (*i.e.*, subclinical PTSD); scores of 47 and higher indicate severe symptoms of PTSD that can possibly fulfil the criteria for PTSD. Psychometric properties of the questionnaire proved to be satisfactory in a sample of four Dutch groups of children after violence and disaster [32]. The internal consistency (Cronbach's alpha) was good (0.92). Convergent validity was high, the CRTI correlated strongly with the Children's Impact of Event Scale (CRIES) (r = .81) [32]. In the present study, the internal consistency reached α = 0.85 (after three month follow-up) and α = 0.89 (after nine month follow-up).

Posttraumatic stress in parents was measured with the Self-Rating Scale for Post Traumatic Stress Disorder (SRS-PTSD) [33,34]. This is a Dutch self-report questionnaire, and contains 17 items corresponding to the diagnostic DSM-IV symptoms of PTSD, divided into three clusters: intrusions (five items), avoidance (seven items), and hyperarousal (five items). With use of this questionnaire, the diagnosis of PTSD and a total symptom score were calculated. The diagnosis of PTSD is likely if at least one intrusion, three avoidance and two hyperarousal symptoms were present in the previous four weeks [2,34]. Furthermore, a total symptom score was calculated by counting all symptoms of PTSD. This continuous scale ranges from 0 (no symptoms at all) to 17 (all symptoms present). The SRS-PTSD demonstrated adequate psychometric properties in a sample of air crash survivors [34]. In general, the clinical utility or validity, and reliability were satisfactory. The sensitivity and specificity were sufficient compared to structured interviews (86% and 80%, respectively). The instrument was regarded as a good alternative to the structured interview for PTSD, particularly at sites that have limited clinical resources [33,34]. In the present study, the internal consistency reached α = 0.91 (after three month follow-up) and α = 0.92 (after nine month follow-up).

Psychological distress in parents was measured using the General Health Questionnaire-30 (*GHQ-30*) [35,36]. The total scale score (0 – 30) can be used as an overall index of psychological distress, for which higher scores indicate greater distress. According to Goldberg et al. [35] scores of 5 or more indicate clinically elevated levels of psychological distress. The validity of the 30-item version is well documented and its internal consistency is highly satisfactory [35,36]. In the present study, the internal consistency reached α = 0.95 (after three month follow-up) and α = 0.94 (after nine month follow-up).

Medical data were obtained from patient records and the Patient Data Management System (PDMS). These data included the following: gender and age of the child; length of stay in PICU; length of ventilatory support; risk of mortality; reason for admission and treatment characteristics. The risk of mortality was measured with the Paediatric Index of Mortality (PIM2). This is a rating index developed to predict mortality risk in the PICU [37]. Reason for admission was categorized by an intensivist at PICU in [1] trauma and [2] non-trauma related admissions. Non-trauma related admission included respiratory insufficiency (27.6%), circulatory insufficiency (51.7%), neurological disorder (17.2%) and metabolic disorder (3.5%).

### Data analysis

The Statistical Package for Social Sciences (SPSS), Windows version 12.0, was used for all analyses. First, missing values were handled according to the guidelines given in the manuals for the relevant questionnaires. Data were imputed if children and parents completed at least 90 percent of the questionnaire by mean scores of the other items. Second, Mann-Whitney tests and Chi-square tests were completed to compare participants and non-participants with regard to patient characteristics. A third analysis examined the prevalence of subclinical PTSD in children using frequency tables. Fourth, a Wilcoxon signed rank test was used to evaluate changes in PTSD scores in children over time. Fifth, we assessed the differences between our study group at nine month follow-up and the Volendam fire disaster group at ten month follow-up. We compared demographics (gender and age) with Mann-Whitney U tests and Chi-square tests, symptoms of PTSD with ANOVA, and subclinical PTSD with logistic regression analyses. Sixth, a correlation matrix was calculated to assess the association between the risk factors and PTSD scores in children at three and nine months. Spearman's rank correlation coefficients were used because of the relatively small numbers of children and lack of normal distribution in most risk factors. The analyzed risk factors included the following: characteristics of the medical event (length of stay in PICU, length of ventilatory support, main reason for PICU admission, risk of mortality and time at follow-up); characteristics of the child (gender, age of the child and PTSD scores at three month follow-up); and characteristics of parental stress reactions at three and nine month follow-up (PTSD and psychological distress in mothers and fathers). The final statistical analysis entered the risk factors that correlated significantly with symptoms of PTSD into a regression analysis. The Backward method was used until, ultimately, a significant model (p < 0.05) including the pertinent risk factors that predict symptoms of PTSD was chosen. The model was tested for linearity. A significance level of 0.05 was used for all tests.

## Results

### Prevalence and course of PTSD

PTSD data were available for 36 children; 21 completed questionnaires at three and nine months follow-ups; eight only completed questionnaires at the three month follow-up; seven only completed questionnaires at the nine month follow-up. Data analyses at the three month follow-up showed that 10 out of 29 (34.5%) children had at least subclinical levels of PTSD. Of these 10 children, four (13.8%) were likely to meet criteria for PTSD. At the nine month follow-up, 10 out of 28 (35.7%) children had at least subclinical levels of PTSD. Of these 10 children, five (17.9%) were likely to meet criteria for PTSD. No statistically significant changes over time were found for symptoms of PTSD (n = 21, z = -0.725, n-ties = 19, p = 0.468). Remarkably, 13 children that scored normal at the three month follow-up also scored normal at the nine month follow-up. Moreover, six children that scored subclinical at the three month follow-up also scored in the subclinical range at the nine month follow-up. Only two children switched scores (one normal to subclinical and one subclinical to normal).

### Prevalence of symptoms of PTSD compared to the Volendam fire disaster

ANOVA was performed to compare the prevalence of symptoms of PTSD between PICU children and children who survived a major fire disaster in the Netherlands. In this ANOVA we used gender and age as covariates because the children, across groups, differed significantly on these two factors. The PICU children were mostly girls (χ^2 ^= 4.47, df = 1, p = 0.035) and were younger (U = 3154.00, n_1 _= 355, n_2 _= 28, p = 0.001) (Table 2). A significant model emerged for symptoms of PTSD (F_(28,355) _= 26.46, p < 0.000, adjusted R^2 ^= 0.21), with an interaction (gender × age, p < 0.000) effect, as well as a main effect for gender (F = 9.96, p = 0.002) and a main effect for age (F = 11.70, p = 0.001). There was no effect for group (F = 0.90, p = 0.343). PICU children and Volendam fire disaster children had the same number of symptoms of PTSD. In addition, the interaction effect indicated that older girls had more symptoms of PTSD than younger girls. A different pattern emerged in boys: Younger boys had more symptoms of PTSD than older boys.

**Table 2:** Demographics and post traumatic stress scores in children at nine months follow-up after paediatric intensive care treatment and Volendam fire disaster.

	Paediatric Intensive Care (n = 28)	Volendam disaster (n = 355)
Gender (girl/boy)	20/8*	180/175
Age (years) (M, (SD))	13.4 (2.6)**	15.2 (1.7)
Symptoms of PTSD (M, (SD))	36.5 (8.1)	38.6 (8.8)
Subclinical PTSD (n, (%))	10 (35.7)	166 (46.4)
PTSD (n, (%))	5 (17.9)	68 (19.0)

*p < 0.05. ** p < 0.01.

PTSD = Post Traumatic Stress Disorder

*Note*: total score of symptoms of PTSD ranges from 26 to 78; subclinical PTSD ≥ 38; PTSD ≥ 47

### Prevalence of PTSD compared to the Volendam fire disaster

Furthermore, logistic regression models for both subclincal PTSD and PTSD corrected for gender, age, and gender × age were performed. These models produced no significant odds ratios for group (PICU children versus Volendam fire disaster children) on either subclinical PTSD (OR = 0.58, 95% CI 0.24 – 1.42, p = 0.231) or PTSD (OR = 0.99, 95% CI 0.33 – 2.97, p = 0.982) (Table 2).

### Correlations between risk factors and symptoms of PTSD

Correlations were calculated between the risk factors and symptoms of PTSD in children at three and nine month follow-ups after paediatric intensive care treatment (Table 3). Significant correlations at the three month follow-up were found between mother's psychological distress score, mother's PTSD score, and child's PTSD score. At the nine month follow-up mother's psychological distress score, father's psychological distress score, mother's PTSD score, as well as child's PTSD score from three month follow-up significantly correlated with symptoms of PTSD of the child at the nine month follow-up. No significant associations were found between characteristics of the medical event and characteristics of the child with PTSD scores at three and nine month follow-ups.

**Table 3:** Correlations between the risk factors and symptoms of PTSD in children at three and nine months follow-up after paediatric intensive care treatment.

	Symptoms of PTSD at three months		Symptoms of PTSD at nine months	
	n	r	n	R

Characteristics of the medical event				
Length of stay in PICU	29	-0.12	28	0.25
Length of artificial ventilation	18	-0.20	20	0.28
Main reason for PICU admission	29	-0.00	28	0.15
Risk of mortality	29	0.11	28	0.06
Follow-up time	29	0.00	28	-0.01

Characteristics of the child				
Gender	29	0.13	28	0.17
Age of the child	29	0.02	28	0.31
Symptoms of PTSD at three months	-	-	21	0.77**

Parental stress reactions at nine months follow-up				
Mother's psychological distress score	25	0.48*	25	0.52**
Mother's PTSD score	27	0.64**	27	0.73**
Father's psychological distress score	23	0.37	21	0.70**
Father's PTSD score	19	0.43	22	0.37

Total	29		28	

*p < 0.05. ** p < 0.01.

PTSD = Post Traumatic Stress Disorder

PICU = Paediatric Intensive Care Unit

### Prediction of symptoms of PTSD at nine month follow-up

Linear regression analysis for symptoms of PTSD at nine month follow-up produced a model with two significant risk factors: (1) mother's PTSD score and (2) child's PTSD score at three month follow-up (R square = 0.818, F = 26.927, p < 0.000). Children reporting more symptoms of PTSD at nine month follow-up had mothers with higher PTSD scores (β = 1.398, t = 4.095, p = 0.001) and had more symptoms of PTSD at three month follow-up (β = 0.383, t = 2.774, p = 0.017). Gender, age, and gender × age were not significant (p < 0.05) factors for symptoms of PTSD in PICU children at nine month follow-up.

## Discussion

The present study shows that, first, over one third of the children older than eight years had subclinical PTSD three months after PICU discharge; one out of seven children were likely to meet criteria for PTSD. Interestingly, PTSD scores did not change over time. Second, PTSD scores after paediatric intensive care treatment are comparable to PTSD scores after a major fire disaster in the Netherlands. Third, the findings of this study illustrate that parental stress reactions (particularly, the mother's) appeared to be the most important indicator for development of PTSD in children compared to the nature of the medical event and the characteristics of the child.

The high prevalence of PTSD in PICU patients is consistent with previous research. Over 10% of children experience stress symptoms to a marked and significant extent after intensive care treatment. Also, in adult intensive care unit (ICU) survivors, PTSD clearly occurs [27,38]. However, exact PTSD prevalence is difficult to determine due to methodological limitations, such as method and timing of PTSD assessment. In a recent observational study in Europe, the prevalence of PTSD in ICU survivors was 9.2% (3.2% – 14.8) [39]. There seems to be a 1:10 risk for developing PTSD among adults and children after intensive care treatment. Although the majority of the ICU survivors are resilient and do recover without any significant stress symptoms, it is important to identify risk factors of PTSD, and to understand whether the risk can be reduced through preventive interventions.

Interestingly, PTSD scores did not significantly change over time. Similar to these findings, a longitudinal study in a community sample of children showed that PTSD is often a persistent and chronic disorder. Although more than half recovered during follow-up at 3 years, the other half showed no significant remission of PTSD symptoms. New stressful events and avoidance symptoms following the initial stressful event seem to predict a chronic course of PTSD [40]. Contrary to these findings, epidemiological studies on PTSD have shown remarkable remission of symptoms of PTSD in the first months after a stressful event [3]. Similarly, a longitudinal study among rape victims showed that 53% recovered by 3 months and an extra 5% recovered by 9 months [41]. The majority with PTSD symptoms appear to recover within weeks rather than months following a stressful event.

This study also aimed to identify risk factors for the development of PTSD. Once these children are identified, supportive care can be offered at an early stage, aimed at minimizing symptoms of PTSD. The present study shows a strong relationship between parental stress reactions, especially from the mother, and PTSD in the child. But, this relationship does not address the question of causality: Does parental distress lead to distress in the child, or vice versa? Nevertheless, high levels of parental distress and potential influence of parents on child well-being highlight the importance of attending to parental reactions when assessing children. Subsequently, interventions for PTSD in paediatrics should focus on the family [7,21-26].

In contrast with previous findings, we did not find a significant relation between the characteristics of the medical event and the development of PTSD. Earlier results have shown that children who were more severely ill and were admitted for a longer period had a greater risk of developing PTSD and psychiatric symptoms [16-18]. These studies examined risk factors immediately or shortly after discharge from the hospital. Only one study examined these medical risk factors in a longitudinal design [17]. Although in their study illness severity and exposure to invasive procedures were initially identified as risk factors for PTSD at six weeks after discharge, these effects decreased at six months.

This is one of the first studies to compare PTSD in children after a paediatric intensive care treatment with another severe stressful event. PTSD prevalence rates in PICU children after nine months equalled those of survivors of a major fire disaster in Volendam. This is in accordance with earlier findings in which the highest rates of PTSD in children were associated with violent events and sexual trauma, followed second by illness and injury, and third by natural disaster and fire [5]. Adult ICU literature reports that survivors of acute respiratory distress syndrome (ARDS) have significantly more symptoms of PTSD than United Nation soldiers who had experienced prolonged service in Cambodia [42]. The significance of mental health care for children after paediatric intensive care treatment is being emphasized by the resemblance between these stressful events.

Some limitations of the study should be addressed. First, a structured clinical interview can be regarded as the best measurement for PTSD. The use of digital self-reports only gives an indication for the diagnosis of PTSD and cut-off scores should be used with caution. Second, almost all children (> 8 years) included in our study were at risk for possible brain damage. Brain injury may possibly lead to an overestimation of PTSD symptoms because symptoms after brain injury overlap significantly with PTSD symptoms. This includes problems with memory, balance, and concentration, as well as irritability [43]. Third, a considerable number of children were lost to follow-up due to non-response and refusal to participate. Although other follow-up studies in the PICU have had similar response rates, this could have biased our results [12,20,44]. Moreover, relatively more girls and trauma patients participated in the study, which also could have biased our results as girls and trauma patients have an increased risk for development of PTSD [15,19] Fourth, this study only reports on children older than 8 years: This is because there is a lack of validated PTSD questionnaires for younger children. As a consequence, we cannot draw conclusions on younger children, although they also express symptoms of PTSD [1]. Fifth, the small and heterogeneous sample may have led to selection bias. Therefore, we must be cautious in generalizing our results towards acute life-threatening medical events in general. The small number of children could also have led to type II errors in comparison to the Volendam data. Type II error is the error of failing to observe a difference when in truth there is one. Small sample sizes are sufficient to produce this difference only when large differences between groups are expected [45]. Finally, although corrected for in the analysis, the significant gender and age difference between the PICU and Volendam children could have biased the results. With these two major limitations, small sample size and possible selection bias, conclusions are only tentative until findings are replicated in a larger study sample.

## Conclusion

The results of the present study suggest that a considerable number of the children had persistent PTSD after paediatric intensive care treatment. Parental stress reactions were the strongest predictor for child PTSD. Prevention of PTSD is important in order to minimize the profound adverse effects that PTSD can have on children's well-being and future development. In the paediatric population PTSD in children is frequently unnoticed and untreated [46]. The presence of symptoms of PTSD in this population underscores the need for medical staff education in identification of PTSD.

## List of abbreviations

PTSD: Post Traumatic Stress Disorder; PICU: Paediatric Intensive Care Unit; ICU: Intensive Care Unit.

## Competing interests

The authors declare that they have no competing interests.

## Authors' contributions

This study is part of an on-going explorative research program on physical and psychological consequences in children and their parents after an unexpected paediatric intensive care admission.

First author, MB, and second author, HK, work together within their PhD program. MB had primary responsibility for the psychological screening of the families, data collection, data entry, all analyses and writing the manuscript. HK participated in the development of the program, had primary responsibility for the physical examination, and contributed to the writing of the manuscript. This program is an initiative of two departments of the Emma Children's Hospital AMC, Amsterdam. APB is head of the paediatric intensive care unit and the fourth author, BFL, is head of the psychosocial department. Both authors supervised the design and execution of the study, and contributed to the writing of the manuscript. Fifth author, MAG, head research of the psychosocial department participated in the development of the program, supervised this study and the final analyses, and contributed to the writing of the manuscript. All authors read and approved the final manuscript.
